# Association of severe stress with the onset of chronic kidney disease after the Great East Japan Earthquake: the Fukushima Health Management Survey

**DOI:** 10.1007/s10157-025-02795-5

**Published:** 2025-11-24

**Authors:** Sakumi Kazama, Fumikazu Hayashi, Kenichi Tanaka, Shiho Sato, Yuka Ueda, Kanako Okazaki, Tetsuya Ohira, Akira Sakai, Masaharu Maeda, Hirooki Yabe, Mitsuaki Hosoya, Atsushi Takahashi, Hironori Nakano, Masanori Nagao, Michio Shimabukuro, Hitoshi Ohto, Seiji Yasumura, Junichiro J. Kazama

**Affiliations:** 1 Radiation Medical Science Center for the Fukushima Health Management Survey, Fukushima, Japan; 2 Department of General Clinical Medicine, Ohu University School of Dentistry, Koriyama, Japan; 3 Department of Epidemiology, Fukushima Medical University School of Medicine, Fukushima, Japan; 4 Department of Nephrology and Hypertension, Fukushima Medical University School of Medicine, 1 Hikariga-oka, Fukushima, Fukushima 9601295 Japan; 5 Department of Physical Therapy, Fukushima Medical University School of Health Sciences, Fukushima, Japan; 6 Department of Radiation Life Sciences, Fukushima Medical University School of Medicine, Fukushima, Japan; 7 Department of Disaster Psychiatry, Fukushima Medical University School of Medicine, Fukushima, Japan; 8 Department of Mind and Brain Medicine, Fukushima Medical University School of Medicine, Fukushima, Japan; 9 Department of Perinatology and Pediatrics for Regional Medical Support, Fukushima Medical University School of Medicine, Fukushima, Japan; 10 Department of Gastroenterology, Fukushima Medical University School of Medicine, Fukushima, Japan; 11 Department of Diabetology and Endocrinology, Fukushima Medical University School of Medicine, Fukushima, Japan

**Keywords:** Chronic kidney disease, K6 score, Great East Japan Earthquake, Severe stress

## Abstract

**Background:**

In 2011, the Great East Japan Earthquake hit the Futaba District on the northeast coast of Japan, followed by a tsunami and a nuclear power plant accident. In this study, we investigated the impact of post‐earthquake life on the onset of chronic kidney disease (CKD) among the residents of the Futaba District.

**Methods:**

Data on 17,859 residents of the Futaba District (7333 men, 10,526 women; mean age: 61.0 ± 10.2 years; mean follow-up period: 3.42 ± 1.51 years) who underwent health checkups and completed self-administered questionnaires in the Fukushima Mental Health and Lifestyle Survey were analyzed. These residents were confirmed to be CKD-free in 2012. Hence, they were assessed for the onset of CKD from 2013 to 2017.

**Results:**

Univariate analysis results showed significant differences between residents with and without CKD. Differences in age, diabetes mellitus, body mass index (BMI), dyslipidemia, hypertension, hyperuricemia, Kessler 6 Psychological Distress Scale (K6) score, smoking habit, alcohol drinking history, exercise habit, history of job change, history of job loss, and evacuation experience were observed. Multivariate analysis was conducted to adjust for multiple factors, and age, BMI, dyslipidemia, hypertension, hyperuricemia, and K6 score were identified as significant promotional factors for CKD onset.

**Conclusion:**

Among the well-recognized risk factors, severe stress reflected by a high K6 score was established to be correlated with CKD onset among residents originally without CKD. Stress management may be another treatment strategy for treating CKD.

## Introduction

On March 11, 2011, the eastern region of Japan was hit by a massive earthquake of magnitude 9.1, later designated the Great East Japan Earthquake. This earthquake triggered a tsunami immediately after, causing a level-7 major accident at the Fukushima Daiichi Nuclear Power Plant, which forced the evacuation of many of the residents around the plant [1, 2]. Disasters and the corresponding evacuation processes impose not only a direct physical impact but also a significant psychological burden.

Chronic kidney disease (CKD) is a clinical concept characterized by persistent reduction in glomerular filtration rate and/or sustained proteinuria, irrespective of the underlying etiology. In Japan, it has been estimated that approximately one in eight adults has CKD (3); however, more recent expert opinion suggests that the prevalence may have increased to as high as one in five. Although various factors are involved in CKD progression, no cohorts for clinical studies on the effects of psychological factors have been studied.

We conducted longitudinal analyses of data from the health checkups of the residents of the Futaba District [3]. This study comprised a cohort of individuals who share an experience with considerable psychological and social impacts. This study aimed to elucidate the impact of post-earthquake life on the onset of CKD, with a focus on the psychological impacts of the earthquake.

## Materials and methods

### Participants

**Fig. 1:**
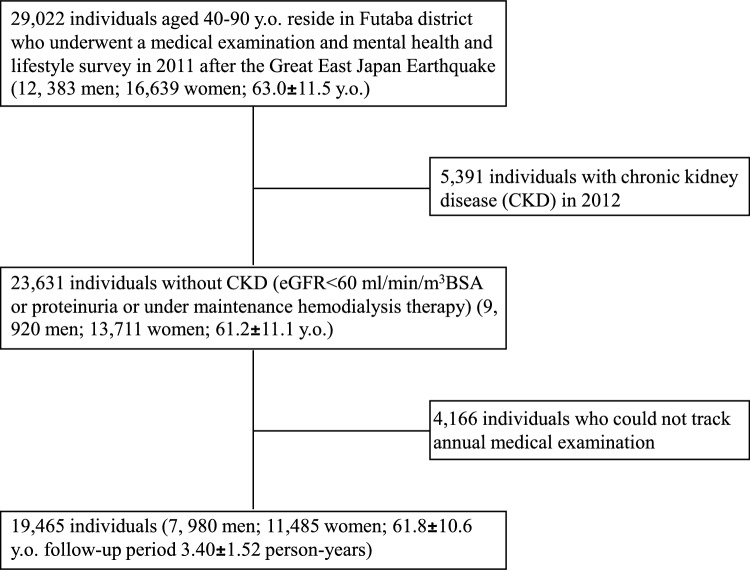
Flowchart of participant inclusion in the analysis

Among the residents of the 13 municipalities who were evacuated during the disaster, 26,619 residents aged 40–90 years participated in the study. These individuals had undergone the Comprehensive Health Check [4] included in the scope of the Fukushima Health Management Survey [5] and completed the self-administered questionnaires in the Mental Health and Lifestyle Survey [6] (valid response count: 73,569 residents; response rate: 40.7%) in 2012. Among them, 17,898 residents who were confirmed to have no CKD in 2012 were assessed for CKD onset from 2013 to 2017. The questionnaires were distributed each February; therefore, the baseline data were obtained from the first survey conducted in February 2012, approximately 11 months after the disaster. We excluded 39 residents lost to follow-up before 2017. Thus, the present study included 17,859 residents in the analysis of the risk factors associated with the onset of CKD (Fig. 1).

### Definition of CKD

eGFR was calculated for each sex based on serum creatinine levels and age using the following formulas recommended by the Japanese Society of Nephrology [7]. In this study, individuals were defined as having CKD if they met any of the following criteria: an eGFR < 60 mL/min/1.73 m^2^; a proteinuria level of ≥ 1 +; or a self-reported history of receiving treatment for chronic renal failure (including maintenance hemodialysis) on the questionnaire.

### Definition of lifestyle diseases

Obesity: body mass index (BMI) ≥ 25 kg/m^2^; hypertension: systolic blood pressure ≥ 140 mmHg, diastolic blood pressure ≥ 90 mmHg, or individuals taking antihypertensives; diabetes mellitus: fasting blood glucose level ≥ 126 mg/dL, hemoglobin A1c (HbA1c) level ≥ 6.5%, or individuals taking hypoglycemics; dyslipidemia: high-density lipoprotein cholesterol level < 40 mg/dL, low-density lipoprotein cholesterol level ≥ 140 mg/dL, fasting triglyceride level ≥ 150 mg/dL, or individuals taking antihyperlipidemic; abnormal hepatic function: aspartate aminotransferase level ≥ 31 U/L, alanine aminotransferase level ≥ 31 U/L, or gamma-glutamyl transferase level ≥ 51 U/L; and hyperuricemia: uric acid level ≥ 7.9 mg/dL and ≥ 5.6 mg/dL for men and women, respectively.

### Definition of psychological distress

The Kessler 6 Psychological Distress Scale (K6) score [8] was adopted to assess psychological distress. The reliability of the Japanese version of the K6 score has been verified [9]. Based on previous studies [10], a K6 score of ≥ 13 was determined to indicate psychological distress.

### Definition of lifestyle

Smoking habit was defined as follows: never smoked, former smoker, or current smoker. Alcohol drinking habits were defined as never drank, former drinker, or current drinker. Sleep satisfaction was classified as satisfied, slightly unsatisfied, unsatisfied, or dissatisfied. Exercise habits were classified as daily, 2 to 4 times a week, once a week, or never.

### Definition of socioeconomic factors

We questioned residents on whether they changed or lost their jobs after the earthquake.

### Definition of evacuation experience

Regarding questions on residence after the earthquake, the residents of the municipalities that were partially designated as evacuation zones were divided into two groups. They included a non-evacuee group comprising residents who stayed in places other than temporary housing or shelters in 2011 and an evacuee group comprising other residents.

### Statistical methods

SAS 9.4 software (SAS Institute Inc., Cary, NC) was employed for statistical analyses. Data were expressed as numbers, mean and standard deviation, or percentage. To identify the risk factors for CKD, hazard ratios and 95% confidence intervals for the association between each factor and the incidence of CKD were obtained from crude, age- and sex-adjusted, and multivariate Cox regression analyses. A *p* value of < 0.05 for a two-tailed test was considered to indicate a significant difference.

## Results

During the follow-up, 4,294 residents (1,782 men and 2,512 women) newly met the criteria for CKD onset. The eGFR levels decreased to < 60 mL/min/1.73 m^2^ in 1,562 men and 2,360 women, and proteinuria was detected in 324 men and 204 women. The clinical features of these residents are shown in Table 1.

**Table 1:** Clinical features of the study participants at the beginning of the observation

	All	Incident CKD	No CKD
	*N* = 17,859	*N* = 4,294	*N* = 13,565
Follow-up period (years)	3.4(1.5)	2.2(1.3)	3.8(1.3)
Age (years)	61(10.2)	65.1(9.2)	59.7(10.1)
Height (cm)	157.4(8.7)	156.3(8.7)	157.7(8.7)
Weight (kg)	59(10.9)	59.2(10.9)	58.9(10.9)
BMI (kg/m^2^)	23.7(3.4)	24.1(3.4)	23.6(3.4)
eGFR (mL/min/1.73 m^2^)	76(11)	67.6(7.1)	78.6(10.6)
FGLU (mg/dL)	101.4(21)	102.9(22.3)	101(20.5)
HbA1c (NGSP)(%)	5.5(0.7)	5.6(0.7)	5.5(0.7)
HDL-chol (mg/dL)	60.8(15.2)	59(14.9)	61.3(15.2)
LDL-chol (mg/dL)	127.1(32)	126.5(32)	127.3(32)
TG (mg/dL)	113.5(74.6)	119.2(71.3)	111.6(75.6)
AST (U/L)	24.9(14.4)	25.7(11.3)	24.6(15.2)
ALT (U/L)	23.2(19.8)	23.3(17)	23.2(20.6)
γ-GT (U/L)	36.2(47.7)	35.6(46.6)	36.4(48)
UA (mg/dL)	4.9(1.3)	5.1(1.3)	4.8(1.3)
SBP (mmHg)	130.9(15.9)	133.6(15.6)	130.1(15.9)
DBP (mmHg)	78.7(10.1)	79.3(10)	78.5(10.2)

Data are shown as mean (SD)

*SD* standard deviation, *CKD* chronic kidney disease, *BMI* body mass index, *eGFR* estimated glomerular filtration rate, *FGLU* fasting plasma glucose, *HbA1c* hemoglobin A1c, *NGSP* National Glycohemoglobin Standardization Program, *HDL-chol* high-density lipoprotein cholesterol, *LDL-chol* low-density lipoprotein cholesterol, *TG* triglyceride, *AST* aspartate aminotransferase, *ALT* alanine transaminase, *γ-GT* γ-glutamyl transferase, *UA* uric acid, *SBP* systolic blood pressure, *DBP* diastolic blood pressure

Univariate analyses performed with the CKD onset as the objective variable showed significant differences between residents with and without CKD. These differences were observed in age at enrollment, diabetes mellitus, BMI, dyslipidemia, hypertension, hyperuricemia, K6 score, smoking history, alcohol drinking history, exercise habit, history of job change, history of job loss, and evacuation experience (Table 2).

**Table 2:** Univariate analyses comparing factors in residents who were affected and unaffected by CKD

Factor		All (%)	Incident CKD (%)	No CKD(%)	*p* value
Age	< 65	11,342(63.5)	2014(46.9)	9328(68.8)	< 0.0001
	≥ 65	6517(36.5)	2280(53.1)	4237(31.2)	
Sex	Men	7333(41.1)	1782(41.5)	5551(40.9)	0.502
	Women	10,526(58.9)	2512(58.5)	8014(59.1)	
Diabetes mellitus	No	15,893(89.2)	3740(87.2)	12,153(89.8)	< 0.0001
	Yes	1922(10.8)	549(12.8)	1373(10.2)	
Obesity	BMI < 25.0	12,058(67.6)	2714(63.2)	9344(68.9)	< 0.0001
	BMI ≥ 25.0	5791(32.4)	1578(36.8)	4213(31.1)	
Dyslipidemia	No	6650(39.4)	1437(35.1)	5213(40.8)	< 0.0001
	Yes	10,211(60.6)	2654(64.9)	7557(59.2)	
Hepatic disorder	No	12,523(70.1)	2986(69.5)	9537(70.3)	0.339
	Yes	5336(29.9)	1308(30.5)	4028(29.7)	
Hypertension	No	8888(49.8)	1683(39.3)	7205(53.2)	< 0.0001
	Yes	8948(50.2)	2604(60.7)	6344(46.8)	
Hyperuricemia	No	16,730(93.7)	3904(90.9)	12,826(94.6)	< 0.0001
	Yes	1129(6.3)	390(9.1)	739(5.4)	
K6 score	< 13	14,227(85.1)	3283(83.5)	10,944(85.6)	0.001
	≥ 13	2485(14.9)	648(16.5)	1837(14.4)	
Smoking habit	Never	10,779(62.2)	2631(63.6)	8148(61.8)	< 0.0001
	Former	4027(23.2)	1052(25.4)	2975(22.6)	
	Current	2519(14.5)	454(11)	2065(15.7)	
Alcohol drinking habits	Never	8805(50.3)	2226(53.2)	6579(49.3)	< 0.0001
	Former	448(2.6)	134(3.2)	314(2.4)	
	Current	8266(47.2)	1825(43.6)	6441(48.3)	
Level of sleep satisfaction	Satisfied	4882(33.4)	1147(33)	3735(33.6)	0.553
	Slightly unsatisfied	6754(46.3)	1616(46.5)	5138(46.2)	
	Unsatisfied	2304(15.8)	540(15.5)	1764(15.9)	
	Dissatisfied	656(4.5)	170(4.9)	486(4.4)	
Exercise habit	Daily	2966(17)	810(19.4)	2156(16.2)	< 0.0001
	2 to 4 times a week	4286(24.6)	1263(30.3)	3023(22.7)	
	Once a week	2652(15.2)	663(15.9)	1989(15)	
	Never	7553(43.3)	1432(34.4)	6121(46.1)	
Job change	No	17,366(97.2)	4213(98.1)	13,153(97)	< 0.0001
	Yes	493(2.8)	81(1.9)	412(3)	
Loss of job	No	13,501(75.6)	3314(77.2)	10,187(75.1)	0.006
	Yes	4358(24.4)	980(22.8)	3378(24.9)	
Evacuation experience	No	7874(44.3)	1809(42.3)	6065(44.9)	0.002
	Yes	9906(55.7)	2472(57.7)	7434(55.1)	

Data are shown as numbers (percentages). Differences between the no CKD and incident CKD groups were analyzed using Chi-squared test

*CKD* chronic kidney disease, *BMI* body mass index

The age‐ and sex‐adjusted Cox regression analysis identified age, BMI, diabetes mellitus, dyslipidemia, hypertension, hyperuricemia, K6 score, smoking history, alcohol drinking history, and exercise habit as significant factors. The multivariate analysis identified age, BMI, dyslipidemia, hypertension, hyperuricemia, and K6 score as significant factors. In addition, alcohol drinking history and exercise habits were identified as significant inhibitory factors (Table 3).

**Table 3:** Cox regression multivariate analysis to identify factors associated with the onset of CKD

Factor	Age‐ and sex‐adjusted HR (95%CI)^a^	Multivariable‐adjusted HR (95%CI)^b^
Older age (ref. AGE < 65)	2.29(2.16–2.43)	1.96(1.84–2.10)
Male sex (Ref. Female)	0.96(0.91–1.02)	1.02(0.94–1.12)
Diabetes mellitus (Ref. No)	1.18(1.08–1.29)	1.09(0.99–1.19)
Obesity (Ref. BMI < 25.0)	1.26(1.19–1.35)	1.12(1.05–1.20)
Dyslipidemia (Ref. No)	1.20(1.12–1.28)	1.11(1.04–1.19)
Hypertension (Ref. No)	1.40(1.31–1.49)	1.30(1.22–1.39)
Hyperuricemia (Ref. No)	1.68(1.51–1.86)	1.56(1.40–1.73)
K6 score (Ref. < 13)	1.14(1.05–1.24)	1.13(1.04–1.23)
Smoking habit (Ref. Never)		
Former	1.09(0.99–1.19)	1.09(1.00–1.20)
Current	0.87(0.78–0.97)	0.91(0.81–1.02)
Alcohol drinking habit (Ref. Never)		
Former	1.09(0.91–1.31)	1.03(0.86–1.23)
Current	0.87(0.81–0.93)	0.85(0.79–0.92)
Exercise habit (Ref. Daily)		
2 to 4 times a week	1.11(1.02–1.21)	1.09(1.00–1.19)
Once a week	1.01(0.91–1.12)	1.00(0.90–1.11)
Never	0.89(0.82–0.98)	0.89(0.82–0.98)
Job change (Ref. No)	0.90(0.72–1.13)	0.98(0.78–1.22)
Loss of job (Ref. No)	1.00(0.93–1.08)	0.98(0.91–1.06)
Evacuation experience (Ref. No)	1.05(0.99–1.12)	1.03(0.97–1.10)

^a^Analysis adjusted by age and sex

^b^Analysis adjusted by age, sex, diabetes mellitus, obesity, dyslipidemia, hypertension, hyperuricemia, K6 score, smoking habit, alcohol drinking habit, exercise habit, job change, loss of job, and evacuation experience

Cox proportional hazard model, *p* < 0.05 was considered statistically significant

*95% CI* 95% confidence interval, *HR* hazard ratio, *Ref* reference, *CKD* chronic kidney disease, *BMI* body mass index, *K6* Kessler psychological distress scale

When the objective variable was set to a new decrease characterized by an eGFR level < 60 mL/min/1.73 m^2^, the multivariate analysis identified age, BMI, dyslipidemia, hypertension, hyperuricemia, and K6 score as significant promotional factors. Further, smoking habit, alcohol drinking habit, and exercise habit were identified as significant inhibitory factors (Table 4).

**Table 4:** Cox regression multivariate analysis to identify factors associated with the onset of CKD indicated by eGFR < 60 mL/min/1.73m^3^

Factor	Age‐ and sex‐adjusted HR (95%CI)^a^	Multivariable‐adjusted HR (95%CI)^b^
Older age (Ref. AGE < 65)	2.47(2.32–2.63)	2.08(1.94–2.23)
Male sex (Ref. Female)	0.90(0.84–0.96)	0.96(0.88–1.05)
Diabetes mellitus (Ref. No)	1.08(0.99–1.19)	0.99(0.90–1.09)
Obesity (Ref. BMI < 25.0)	1.25(1.17–1.33)	1.11(1.04–1.19)
Dyslipidemia (Ref. No)	1.18(1.10–1.26)	1.10(1.03–1.18)
Hypertension (Ref. No)	1.41(1.32–1.51)	1.33(1.24–1.42)
Hyperuricemia (Ref. No)	1.69(1.52–1.88)	1.57(1.41–1.75)
K6 score (Ref. < 13)	1.12(1.02–1.22)	1.12(1.02–1.22)
Smoking habit (Ref. Never)		
Former	1.09(0.99–1.20)	1.10(1.00–1.20)
Current	0.79(0.70–0.89)	0.83(0.74–0.93)
Alcohol drinking habit (Ref. Never)		
Former	1.19(0.99–1.43)	1.13(0.94–1.35)
Current	0.87(0.81–0.94)	0.86(0.80–0.93)
Exercise habit (Ref. Daily)		
2 to 4 times a week	1.09(1.00–1.20)	1.08(0.98–1.18)
Once a week	1.01(0.91–1.12)	1.00(0.90–1.11)
Never	0.87(0.79–0.95)	0.87(0.79–0.96)
Job change (Ref. No)	0.84(0.66–1.07)	0.92(0.72–1.17)
Loss of job (Ref. No)	0.98(0.91–1.06)	0.97(0.90–1.05)
Evacuation experience (Ref. No)	1.02(0.96–1.09)	1.01(0.95–1.08)

^a^Analysis adjusted by age and sex

^b^Analysis adjusted by age, sex, diabetes mellitus, obesity, dyslipidemia, hypertension, hyperuricemia, K6 score, smoking habit, alcohol drinking habit, exercise habit, job change, loss of job, and evacuation experience

Cox proportional hazard model, *p* < 0.05 was considered statistically significant

*95% CI* 95% confidence interval, *HR* hazard ratio, *Ref* reference, *eGFR* estimated glomerular filtration rate, *BMI* Body mass index, *K6* Kessler psychological distress scale

Finally, when the objective variable was set to the onset of proteinuria, the multivariate analysis identified age, sex, diabetes mellitus, BMI, hypertension, hyperuricemia, and smoking habit as significant promotional factors (Table 5).

**Table 5:** Cox regression multivariate analysis to identify factors associated with the onset of proteinuria

Factor	Age‐ and sex‐adjusted HR (95%CI)^a^	Multivariable‐adjusted HR (95%CI)^b^
Older age (Ref. AGE < 65)	1.66(1.44–1.92)	1.50(1.27–1.76)
Male sex (Ref. Female)	2.32(2.00–2.69)	1.81(1.47–2.23)
Diabetes mellitus (Ref. No)	2.55(2.17–3.01)	2.28(1.93–2.69)
Obesity (Ref. BMI < 25.0)	1.64(1.42–1.89)	1.37(1.18–1.59)
Dyslipidemia (Ref. No)	1.27(1.08–1.48)	1.07(0.91–1.26)
Hypertension (Ref. No)	1.89(1.61–2.23)	1.62(1.37–1.92)
Hyperuricemia (Ref. No)	1.60(1.27–2.01)	1.37(1.09–1.73)
K6 score (Ref. < 13)	1.16(0.94–1.43)	1.11(0.90–1.37)
Smoking habit (Ref. Never)		
Former	1.34(1.09–1.64)	1.30(1.06–1.59)
Current	1.74(1.39–2.18)	1.83(1.45–2.30)
Alcohol drinking habit (Ref. Never)		
Former	1.02(0.69–1.49)	0.93(0.64–1.37)
Current	0.93(0.79–1.10)	0.89(0.75–1.05)
Exercise habit (Ref. Daily)		
2 to 4 times a week	1.23(1.00–1.52)	1.19(0.96–1.46)
Once a week	0.96(0.74–1.24)	0.94(0.72–1.21)
Never	1.08(0.88–1.34)	1.04(0.84–1.29)
Job change (Ref. No)	0.96(0.57–1.60)	1.03(0.61–1.72)
Loss of job (Ref. No)	1.20(1.02–1.42)	1.12(0.94–1.33)
Evacuation experience (Ref. No)	1.17(1.01–1.35)	1.08(0.93–1.26)

^a^Analysis adjusted by age and sex

^b^Analysis adjusted by age, sex, diabetes mellitus, obesity, dyslipidemia, hypertension, hyperuricemia, K6 score, smoking habit, alcohol drinking habit, exercise habit, job change, loss of job, and evacuation experience

Cox proportional hazard model, *p* < 0.05 was considered statistically significant

*95% CI* 95% confidence interval, *HR* hazard ratio, *Ref* Reference, *BMI* body mass index, *K6* Kessler psychological distress scale

To clarify the impact of psychological stress on the onset of chronic CKD, participants were divided into two groups based on their K6 scores: ≥ 13 and < 13. There were no significant differences in baseline characteristics that might influence CKD development between these groups (Table 6). Furthermore, the group with a K6 score ≥ 13 showed a significantly greater annual decline in eGFR—defined as (baseline eGFR–most recent eGFR) divided by the follow-up period—compared to the group with a K6 score < 13, even after adjustment for multiple confounding variables (Table 7).

**Table 6:** Baseline characteristics of participants according to K6 score category

	K6 < 13	≥ 13	*p* value
	*N* = 14,227	*N* = 2,485	
Follow-up period (years)			
Age (years)	60.6 (10.1)	60.6(10.0)	0.893
Height (cm)	157.9 (8.6)	156.2 (8.4)	< 0.0001
Weight (kg)	59.4 (10.9)	57.8 (11.0)	< 0.0001
BMI (kg/m^2^)	23.7 (3.4)	23.6 (3.6)	0.086
eGFR (mL/min/1.73 m^2^)	76.1 (11.1)	76.0 (10.6)	0.609
FGLU (mg/dL)	101.5 (20.8)	100.6 (22.0)	0.081
HbA1c (NGSP)(%)	5.5 (0.7)	5.5 (0.7)	0.571
HDL-chol (mg/dL)	60.8 (15.2)	61.1 (15.2)	0.365
LDL-chol (mg/dL)	127.4 (31.8)	126.5 (32.8)	0.239
TG (mg/dL)	113.5 (75.0)	114.1 (78.5)	0.723
AST (U/L)	24.9 (13.9)	24.6 (17.6)	0.512
ALT (U/L)	23.4 (20.3)	22.9 (18.4)	0.216
γ-GT (U/L)	37.0 (48.7)	33.7 (45.7)	0.001
UA (mg/dL)	4.9 (1.3)	4.7 (1.2)	< 0.0001
SBP (mmHg)	130.9 (15.9)	129.9 (16.0)	0.003
DBP (mmHg)	78.8 (10.2)	78.0 (10.2)	0.0006

Data are shown as mean (SD)

*SD* standard deviation, *CKD* chronic kidney disease, *BMI* body mass index, *eGFR* estimated glomerular filtration rate, *FGLU* fasting plasma glucose, *HbA1c* hemoglobin A1c, *NGSP* National Glycohemoglobin Standardization Program, *HDL-chol* high-density lipoprotein cholesterol, *LDL-chol* low-density lipoprotein cholesterol, *TG* triglyceride, *AST* aspartate aminotransferase, *ALT* alanine transaminase, *γ-GT* γ-glutamyl transferase, *UA* uric acid, *SBP* systolic blood pressure, *DBP* diastolic blood pressure

**Table 7:** Comparison of eGFR decline between high and low K6 score groups

Factor	Parameter estimate	95%CI	Pr >|t|
K6 score (Ref. < 13)	3.681	1.234 to 6.128	0.003

Analysis adjusted by age, sex, diabetes mellitus, obesity, dyslipidemia, hypertension, hyperuricemia, smoking habit, alcohol drinking habit, exercise habit, job change, loss of job, and evacuation experience. Multiple regression analysis, *p* < 0.05 was considered statistically significant

*95% CI* 95% confidence interval

## Discussion

This study included 17,859 residents of the Futaba District, of whom 4644 or approximately 24% developed CKD within 5 years. This incidence rate is slightly higher than that in a previous study involving a cohort of residents with stable living conditions in Japan [11].

Hypertension, diabetes mellitus, smoking habit [12–14], metabolic syndrome [15], and hyperuricemia [16] show positive associations with the onset and progression of CKD, and high alcohol consumption promote CKD [17]. The data in this study were generally consistent with these conclusions. However, the prevalence of hypertension [18], diabetes mellitus [19], and metabolic syndrome [20] is reportedly associated with the evacuation after the Great East Japan Earthquake and the nuclear power plant accident. Thus, they may be indirectly associated with the earthquake disaster. The prevalence of dyslipidemia [21] was confirmed to have increased after the disaster, which may be associated with CKD progression [22]. It is assumed that hepatic dysfunction may be associated with renal disease [23] or that both may have resulted from metabolic syndrome.

This study shows that a high K6 score promotes the onset and progression of CKD. The K6 score is a tool developed for screening mental disorders, and it is widely employed in public surveys as an index for the degree of any mental problem, including psychological stress [8]. Earthquakes induce severe psychological stress in disaster victims [24], as evidenced by the generally high K6 scores observed in the present cohort. In particular, a K6 score ≥ 13 points, considered to reflect mood-disorder levels that cause social dysfunction, was found, for the first time, to be significantly associated with a decrease in eGFR to < 60 mL/min/1.73 m^2^.

A high incidence of depression has been noted in patients with CKD at the pre-dialysis stage [25]. Studies have reported that depression detected by screening with the Beck Depression Inventory is associated with a secondary outcome of an eGFR-level-related decrease in patients with CKD at the pre-dialysis stage [26]. Furthermore, studies on patients with CKD at the pre-dialysis stage [27] or dialysis stage [28] have revealed that depression is associated with a poor prognosis. However, because patients with established CKD were the subjects of these studies, it could not be concluded whether emotional disturbance was a cause or a result of the worse clinical outcome. We examined residents without CKD and derived an association between depression and decreased eGFR levels. To the best of our knowledge, this is the first report of this finding, attributable to the highly specific nature of the cohort (i.e., a public cohort with shared experiences of potentially stressful situations).

A depressive state has been associated with glomerular hyperfiltration [29], possibly providing an important pathophysiological explanation for the association between a high K6 score and decreased eGFR levels. Hypertension has been reported to increase not only after the Great East Japan Earthquake [30] but also after many natural disasters [31]. This type of hypertension, designated disaster hypertension, is considered to mainly arise through the stress-dependent hyperactivation of the sympathetic nervous system [32]. This condition is another possible mediator of renal damage through the contraction of the glomerular afferent artery and the subsequent activation of the renin–angiotensin–aldosterone system that causes glomerular hyperfiltration. Indeed, it has been suggested that at least part of the effect of stress on renal function may be mediated through its influence on blood pressure and glucose metabolism [33]. In addition to its potential direct effects on the kidneys, stress may also contribute to the onset and progression of CKD by altering behavioral patterns, including dietary habits and healthcare-seeking behaviors.

Although many of the residents in the Futaba District were evacuated to avoid radiation exposure, the exposure levels were low not only in the evacuees but also in the residents who did not evacuate [34, 35]. Radiation-induced renal injury, when it occurs, is typically delayed in onset, with hematopoietic, gastrointestinal, and cutaneous toxicities expected to manifest earlier [36]. However, surveys have not identified such radiation-related conditions among residents of the Futaba region. Thus, we consider that radiation is unlikely to have directly affected the changes in renal function in the present study. Thus, we did not consider radiation exposure as a direct cause of CKD in this study. We do not negate the possibility of radiation‐derived health injuries in the future. However, from a scientific standpoint, it is inappropriate to conclude that all ailments afflicting the residents of the Futaba District must be related to radiation exposure.

This study has several limitations. Under standard diagnostic criteria, confirmation of chronic kidney disease (CKD) requires evidence that reduced eGFR or proteinuria persists for at least 3 months. However, in this study, data were available only once annually, and therefore CKD was defined based on a single observation of reduced eGFR or proteinuria. This operational decision was unavoidable given the nature of this cohort, and the same criterion has been used in prior studies utilizing this dataset. Nonetheless, we acknowledge that this approach does not allow exclusion of transient abnormalities and represents a clear limitation of the study. One of the common pathways for CKD progression is glomerular hyperfiltration. In this case, the eGFR level increases transiently and subsequently decreases. This may also be true in individuals suffering from depression [29], as discussed above. Even when some factors adversely affect the kidney through hyperfiltration, a cohort will likely include individuals with both increased and decreased eGFR levels during a longitudinal observation study. Thus, the impact of psychological stress on the kidney may be considerably more severe than that revealed in the present study. A similar bias may also apply to smoking. Nicotine is known to induce glomerular hyperfiltration through the upregulation of COX-2 expression [37], and this hyperfiltration is understood to contribute to long-term renal damage. In fact, some observational studies with short follow-up periods have reported that smoking was associated with the onset of proteinuria, while paradoxically showing higher GFR values in many cases [38]. In the present study as well, smoking appeared to be associated with an increased incidence of proteinuria but a seemingly attenuated decline in renal function. However, this should be interpreted as a consequence of glomerular hyperfiltration, and not as evidence against the nephrotoxic effects of smoking.

This cohort was established after the Great East Japan Earthquake, and therefore lacks pre-disaster data. It is generally recognized that acute elevations in blood pressure frequently occur following natural disasters [32], and particularly after the Great East Japan Earthquake, a pronounced increase in blood pressure was reported among patients with chronic kidney disease [18]. However, this study could not distinguish between individuals with disaster-related acute hypertension and those with preexisting essential hypertension. Similarly, it was not possible to differentiate participants with elevated K6 scores that developed after the earthquake from those who had high scores prior to the disaster. Although the renal consequences may differ between these subgroups, our inability to disentangle them constitutes a limitation of this study.

Other limitations of this study include the use of the dipstick method for proteinuria assessment, which is less reliable than quantitative measurements; the fact that both proteinuria and eGFR were measured only once annually; the fact that information on lifestyle habits was obtained through self-administered questionnaires with unverified accuracy; and that many of the evaluated factors, including the K6 score, were assessed only at baseline. Furthermore, as the response rate to the questionnaire was limited to 40.7%, it is possible that the results do not fully reflect the overall population trends. Another limitation of this study is the absence of data on treatment status for hyperuricemia and liver dysfunction.

However, the present study is still targeted a highly specific cohort within the public that shared an experience with potentially stressful situations. The sample size was sufficiently large. The results revealed that severe stress is associated with decreased eGFR levels. This finding is sufficiently reliable and may contribute to future clinical practices and studies.

In conclusion, a longitudinal analysis of data obtained from the health checkups of the residents of the Futaba District who were severely affected by the disaster was conducted. The results revealed that CKD onset, particularly characterized by a decrease in the eGFR level, is associated with severe stress, in addition to known associated factors. Even more than a decade after the disaster, residents of the Futaba region continue to experience stressful lives due to persistent stigma and discrimination. Evaluating whether stress management can suppress the onset and progression of CKD represents the next challenge to be addressed. If the mechanism by which stress induces renal impairment involves the induction of glomerular hyperfiltration, then stress management may at least have the potential to delay CKD progression. Fortunately, a comprehensive health screening system has been established in the Futaba area following the nuclear accident, enabling continuous monitoring of residents’ health status. In parallel, the regional medical service infrastructure has also been reinforced. Should active stress interventions prove effective, they may offer a novel therapeutic option in the management of CKD.

## Data Availability

The FHMS datasets analyzed during the present study are not publicly available because they belong to the Fukushima Prefecture Government and can only be used within that organization.

## References

[1] Kazama JJ, Narita I. Earthquake in Japan. Lancet. 2011;377:1652–3. 21571143 10.1016/S0140-6736(11)60673-9

[2] Hayashi Y, Nagai M, Ohira T, Satoh H, Sakai A, Ohtsuru A, et al. The impact of evacuation on the incidence of chronic kidney disease after the Great East Japan Earthquake: the Fukushima Health Management Survey. Clin Exp Nephrol. 2017;21:995–1002. 28299459 10.1007/s10157-017-1395-8 PMC5698380

[3] Yasumura S, Hosoya M, Yamashita S, Kamiya K, Abe M, Akashi M, et al. Study protocol for the Fukushima health management survey. J Epidemiol. 2012;22:375–83. 22955043 10.2188/jea.JE20120105 PMC3798631

[4] Ohira T, Nakano H, Okazaki K, Hayashi F, Nagao M, Sakai A, et al. Trends in lifestyle-related diseases and their risk factors after the Fukushima Daiichi nuclear power plant accident: results of the comprehensive health Check in the Fukushima health management survey. J Epidemiol. 2022;32:S36–46. 36464299 10.2188/jea.JE20210386 PMC9703921

[5] Imai E, Horio M, Watanabe T, Iseki K, Yamagata K, Hara S, et al. Prevalence of chronic kidney disease in the Japanese general population. Clin Exp Nephrol. 2009;13:621–30. 19513802 10.1007/s10157-009-0199-x

[6] Maeda M, Harigane M, Horikoshi N, Takebayashi Y, Sato H, Takahashi A, et al. Long-term, community-based approach for affected people having problems with mental health and lifestyle issues after the 2011 Fukushima disaster: the Fukushima health management survey. J Epidemiol. 2022;32:S47–56. 36464300 10.2188/jea.JE20210178 PMC9703932

[7] Matsuo S, Imai E, Horio M, Yasuda Y, Tomita K, Nitta K, et al. Revised equations for estimated GFR from serum creatinine in Japan. Am J Kidney Dis. 2009;53:982–92. 19339088 10.1053/j.ajkd.2008.12.034

[8] Kessler RC, Andrews G, Colpe LJ, Hiripi E, Mroczek DK, Normand SL, et al. Short screening scales to monitor population prevalences and trends in non-specific psychological distress. Psychol Med. 2002;32:959–76. 12214795 10.1017/s0033291702006074

[9] Furukawa TA, Kawakami N, Saitoh M, Ono Y, Nakane Y, Nakamura Y, et al. The performance of the Japanese version of the K6 and K10 in the World Mental Health Survey Japan. Int J Methods Psychiatr Res. 2008;17:152–8. 18763695 10.1002/mpr.257 PMC6878390

[10] Hayashi F, Abe K, Sato M, Ohira T, Sato S, Takahashi A, et al. Trajectories of liver dysfunction and long-term evacuation status after the great East Japan earthquake: the Fukushima Health Management Survey. Int J Disaster Risk Reduct. 2024;108:104513.

[11] Iseki K, Asahi K, Moriyama T, Yamagata K, Tsuruya K, Yoshida H, et al. Risk factor profiles based on estimated glomerular filtration rate and dipstick proteinuria among participants of the specific health check and guidance system in Japan 2008. Clin Exp Nephrol. 2012;16:244–9. 22057582 10.1007/s10157-011-0551-9

[12] Noborisaka Y, Ishizaki M, Yamada Y, Honda R, Yokoyama H, Miyao M, et al. The effects of continuing and discontinuing smoking on the development of chronic kidney disease (CKD) in the healthy middle-aged working population in Japan. Environ Health Prev Med. 2013;18:24–32. 22623223 10.1007/s12199-012-0285-7 PMC3541810

[13] Yoon HJ, Park M, Yoon H, Son KY, Cho B, Kim S. The differential effect of cigarette smoking on glomerular filtration rate and proteinuria in an apparently healthy population. Hypertens Res. 2009;32:214–9. 19262485 10.1038/hr.2008.37

[14] Xia J, Wang L, Ma Z, Zhong L, Wang Y, Gao Y, et al. Cigarette smoking and chronic kidney disease in the general population: a systematic review and meta-analysis of prospective cohort studies. Nephrol Dial Transplant. 2017;32:475–87. 28339863 10.1093/ndt/gfw452

[15] Okada R, Yasuda Y, Tsushita K, Wakai K, Hamajima N, Matsuo S. Trace proteinuria by dipstick screening is associated with metabolic syndrome, hypertension, and diabetes. Clin Exp Nephrol. 2018;22:1387–94. 29934666 10.1007/s10157-018-1601-3

[16] Oh TR, Choi HS, Kim CS, Bae EH, Ma SK, Sung SA, et al. Hyperuricemia has increased the risk of progression of chronic kidney disease: propensity score matching analysis from the KNOW-CKD study. Sci Rep. 2019;9:6681. 31040373 10.1038/s41598-019-43241-3 PMC6491556

[17] Park M, Lee SM, Yoon HJ. Association between alcohol intake and measures of incident CKD: an analysis of nationwide health screening data. PLoS ONE. 2019;14:e0222123. 31539384 10.1371/journal.pone.0222123 PMC6754126

[18] Tanaka K, Nakayama M, Kanno M, Kimura H, Watanabe K, Tani Y, et al. Home blood pressure control after the great East Japan earthquake in patients on chronic hemodialysis. Ther Apher Dial. 2014;18:149–54. 24720405 10.1111/1744-9987.12072

[19] Satoh H, Ohira T, Hosoya M, Sakai A, Watanabe T, Ohtsuru A, et al. Evacuation after the Fukushima Daiichi nuclear power plant accident is a cause of diabetes: results from the Fukushima health management survey. J Diabetes Res. 2015;2015:1–9. 10.1155/2015/627390 PMC4461763 26106625

[20] Takahashi A, Ohira T, Okazaki K, Yasumura S, Sakai A, Maeda M, et al. Effects of psychological and lifestyle factors on metabolic syndrome following the Fukushima Daiichi nuclear power plant accident: the Fukushima health management survey. J Atheroscler Thromb. 2020;27:1010–8. 32009075 10.5551/jat.52225 PMC7508722

[21] Satoh H, Ohira T, Nagai M, Hosoya M, Sakai A, Watanabe T, et al. Hypo-high-density lipoprotein cholesterolemia caused by evacuation after the Fukushima Daiichi nuclear power plant accident: results from the Fukushima health management survey. Intern Med. 2016;55:1967–76. 27477401 10.2169/internalmedicine.55.6030

[22] Tsuruya K, Yoshida H, Nagata M, Kitazono T, Iseki K, Iseki C, et al. Impact of the triglycerides to high-density lipoprotein cholesterol ratio on the incidence and progression of CKD: a longitudinal study in a Large Japanese population. Am J Kidney Dis. 2015;66:972–83. 26145254 10.1053/j.ajkd.2015.05.011

[23] Sharma N, Sircar A, Anders HJ, Gaikwad AB. Crosstalk between kidney and liver in non-alcoholic fatty liver disease: mechanisms and therapeutic approaches. Arch Physiol Biochem. 2022;128:1024–38. 32223569 10.1080/13813455.2020.1745851

[24] Maeda M, Oe M. Mental health consequences and social issues after the Fukushima disaster. Asia Pac J Public Health. 2017;29:36S-46S. 28330398 10.1177/1010539516689695

[25] Palmer S, Vecchio M, Craig JC, Tonelli M, Johnson DW, Nicolucci A, et al. Prevalence of depression in chronic kidney disease: systematic review and meta-analysis of observational studies. Kidney Int. 2013;84:179–91. 23486521 10.1038/ki.2013.77

[26] Tsai YC, Chiu YW, Hung CC, Hwang SJ, Tsai JC, Wang SL, et al. Association of symptoms of depression with progression of CKD. Am J Kidney Dis. 2012;60:54–61. 22495469 10.1053/j.ajkd.2012.02.325

[27] Hedayati SS, Minhajuddin AT, Afshar M, Toto RD, Trivedi MH, Rush AJ. Association between major depressive episodes in patients with chronic kidney disease and initiation of dialysis, hospitalization, or death. JAMA. 2010;303:1946–53. 20483971 10.1001/jama.2010.619 PMC3217259

[28] Kazama S, Kazama JJ, Wakasugi M, Ito Y, Narita I, Tanaka M, et al. Emotional disturbance assessed by the Self-Rating Depression Scale test is associated with mortality among Japanese hemodialysis patients. Fukushima J Med Sci. 2018;64:23–9. 29398691 10.5387/fms.2016-21 PMC5956087

[29] Lin M, Huang H, Yao J, Liang J, Li L, Lin W, et al. Association between depression and renal hyperfiltration in a general Chinese population. Kidney Blood Press Res. 2019;44:1441–52. 31734665 10.1159/000503922

[30] Ohira T, Hosoya M, Yasumura S, Satoh H, Suzuki H, Sakai A, et al. Evacuation and risk of hypertension after the great East Japan earthquake: the Fukushima health management survey. Hypertension. 2016;68:558–64. 27480836 10.1161/HYPERTENSIONAHA.116.07499

[31] Tabrizi JS, Farahbakhsh M, Sadeghi-Bazargani H, Abdolahi HM, Nikniaz Z, Farhangi MA, et al. Health consequences of Lake Urmia in crisis in the disaster area: a pilot study. Disaster Med Public Health Prep. 2020;14:442–8. 31452493 10.1017/dmp.2019.61

[32] Kario K. Disaster hypertension–its characteristics, mechanism, and management–. Circ J. 2012;76:553–62. 22327030 10.1253/circj.cj-11-1510

[33] Koga K, Hara M, Shimanoe C, Nishida Y, Furukawa T, Iwasaka C, et al. Association of perceived stress and coping strategies with the renal function in middle-aged and older Japanese men and women. Sci Rep. 2022;12:291. 34997128 10.1038/s41598-021-04324-2 PMC8742036

[34] Nomura S, Murakami M, Naito W, Yasutaka T, Sawano T, Tsubokura M. Low dose of external exposure among returnees to former evacuation areas: a cross-sectional all-municipality joint study following the 2011 Fukushima Daiichi nuclear power plant incident. J Radiol Prot. 2020;40:1–18. 31809269 10.1088/1361-6498/ab49ba

[35] Nomura S, Oikawa T, Tsubokura M. Low dose from external radiation among returning residents to the former evacuation zone in Minamisoma City, Fukushima Prefecture. J Radiol Prot. 2019;39:548–63. 31013251 10.1088/1361-6498/ab0f87

[36] Dawson LA, Kavanagh BD, Paulino AC, Das SK, Miften M, Li XA, et al. Radiation-associated kidney injury. Int J Radiat Oncol Biol Phys. 2010;76:S108–15. 20171504 10.1016/j.ijrobp.2009.02.089

[37] Rangarajan S, Rezonzew G, Chumley P, Fatima H, Golovko MY, Feng W, et al. COX-2-derived prostaglandins as mediators of the deleterious effects of nicotine in chronic kidney disease. Am J Physiol Renal Physiol. 2020;318:F475–85. 31841390 10.1152/ajprenal.00407.2019 PMC7052654

[38] Maeda I, Hayashi T, Sato KK, Koh H, Harita N, Nakamura Y, et al. Cigarette smoking and the association with glomerular hyperfiltration and proteinuria in healthy middle-aged men. Clin J Am Soc Nephrol. 2011;6:2462–9. 21885794 10.2215/CJN.00700111 PMC3359554

